# Full-Automatic High-Efficiency Mueller Matrix Microscopy Imaging for Tissue Microarray Inspection

**DOI:** 10.3390/s24144703

**Published:** 2024-07-20

**Authors:** Hanyue Wei, Yifu Zhou, Feiya Ma, Rui Yang, Jian Liang, Liyong Ren

**Affiliations:** 1 School of Physics and Information Technology, Shaanxi Normal University, Xi’an 710119, China; whyy24@snnu.edu.cn (H.W.); zhouyf@snnu.edu.cn (Y.Z.); feiyam@snnu.edu.cn (F.M.); yangyr@snnu.edu.cn (R.Y.); jianliang@snnu.edu.cn (J.L.); 2 Xi’an Key Laboratory of Optical Information Manipulation and Augmentation (OMA), Xi’an 710119, China; 3 Robust (Xixian New Area) Opto-Electro Technologies Co., Ltd., Xi’an 712000, China

**Keywords:** Mueller matrix microscopy imaging, cancerous cervical inspection, tissue microarray, polarization measurement

## Abstract

This paper proposes a full-automatic high-efficiency Mueller matrix microscopic imaging (MMMI) system based on the tissue microarray (TMA) for cancer inspection for the first time. By performing a polar decomposition on the sample’s Mueller matrix (MM) obtained by a transmissive MMMI system we established, the linear phase retardance equivalent waveplate fast-axis azimuth and the linear phase retardance are obtained for distinguishing the cancerous tissues from the normal ones based on the differences in their polarization characteristics, where three analyses methods including statistical analysis, the gray-level co-occurrence matrix analysis (GLCM) and the Tamura image processing method (TIPM) are used. Previous MMMI medical diagnostics typically utilized discrete slices for inspection under a high-magnification objective (20×–50×) with a small field of view, while we use the TMA under a low-magnification objective (5×) with a large field of view. Experimental results indicate that MMMI based on TMA can effectively analyze the pathological variations in biological tissues, inspect cancerous cervical tissues, and thus contribute to the diagnosis of postoperative cancer biopsies. Such an inspection method, using a large number of samples within a TMA, is beneficial for obtaining consistent findings and good reproducibility.

## 1. Introduction

Cervical cancer is the most common gynecological malignancy [1,2]. Conventional methods for cervical cancer detection include colposcopy [3], cervical cytology (pap smear) [4], human papilloma virus (HPV) testing [5], and biopsy [6]. However, the colposcopy, the cervical cytology, and the HPV testing methods can only assist in determining cervical abnormalities and cannot provide definitive diagnostic results. The “gold standard” for diagnosing cancerous cervical tissues remains the combination of biopsy and pathological examination. Biopsy can be performed preoperatively [7], intraoperatively [8], or postoperatively [9]. The histopathological diagnosis in the postoperative biopsy process involves three steps: firstly, multiple samples are taken from the entire lesion and its surrounding affected tissues and organs, as well as relevant lymph nodes; secondly, the collected samples are prepared as pathological tissue slices; thirdly, pathologists examine the slices under a microscope to diagnose the condition of the lesion and determine the treatment plan. However, due to the labor-intensive process of preparing multiple pathological tissue slices for postoperative biopsy, it typically takes 3 to 7 days to issue a diagnostic report. Therefore, a new technique is urgently needed to improve the efficiency of postoperative biopsy.

TMA provides a high-throughput histological technique [10,11] that allows multiple tissue samples to be arranged on a single glass slice, creating a small tissue microarray for diagnosis. This technology enables efficient and rapid large-scale histological analysis, significantly shortening the time required for postoperative biopsy diagnosis. Currently, TMA has been widely applied in life sciences and clinical fields, including disease diagnosis and prognostic evaluation [12], drug research and development [12,13], cancer research [14], and more. Common detection methods for TMA include immunohistochemistry [15], in situ hybridization [16], fluorescence in situ hybridization [17], polymerase chain reaction (PCR) [18], and mass spectrometry imaging [19]. However, the immunohistochemistry, the in situ hybridization, and the PCR methods require specific probes. On the other hand, the fluorescence in situ hybridization and the mass spectrometry imaging methods both rely on fluorescent labeling. These techniques have high technical requirements and relatively strict storage conditions, which hinder their widespread adoption. Therefore, it is crucial to develop a new label-free and simplified detection technology for TMA.

Humans have a long history of utilizing light waves for biological microstructure examination. Light waves possess four fundamental properties: intensity, wavelength, phase, and polarization. Although optics-based biosensing has been extensively utilized in clinical diagnostics, polarization, being the last discovered fundamental property of light waves by humans, has only recently garnered significant attention in the biomedical field [20].

The polarization state of light undergoes changes during its propagation in biological tissues, and the polarization information also varies with different microscopic structures within biological tissues. The MM records the complete polarization information of biological tissues, fully reflecting their polarization properties [21]. MMMI [22,23,24], as a label-free, non-contact, and non-invasive imaging technique, reflects the polarization characteristics of the sample by measuring the polarization state changes between the incident and the transmission lights and has been applied in the detection of liver cancer [25], skin cancer [26], breast cancer [27], and others by using their pathological tissue slices.

Considering the high complexity of traditional TMA inspection techniques and the low efficiency of the polarization detection technique, this study proposes a high-efficiency TMA-based cancer inspection method based on MMMI. It is found in the experiment that, by using a modified commercial microscope, the 4 × 4 MM of TMA samples can be acquired, and the equivalent waveplate fast-axis azimuth angle (*θ*) and the phase retardation (*δ*) of the sample can be obtained through MM polar decomposition. In addition, by analyzing the statistical features of the *θ* and *δ* images using kurtosis and skewness and further combining them with the texture feature analysis methods of GLCM and TIPM, the effective diagnosis of cancerous cervical samples on TMA can be achieved. The method, based on the high-throughput nature of TMA and the MMMI technique, enables high-throughput, label-free, non-invasive, and non-contact cancer detection. It should be pointed out that, based on many previously reported research works on tissue slice detection, the above two polarization parameters are effective by using a high-magnification objective (20×–50×) under high-resolution imaging with a small field of view; in our cases, when using the TMA, it is found that they are also effective where a low-magnification objective (5×) is used. Although high-magnification objectives (20×–50×) capture more detailed tissue information due to their high resolution and small field of view for providing more precise tissue identification, this high-resolution imaging is less efficient. It requires using a low-magnification objective first to identify abnormal tissue and then switching to a high-magnification objective for detailed observation. This paper uses a low-magnification objective (5×), which, despite its lower resolution, offers a larger field of view, facilitating rapid detection.

In this study, we constructed a fully automated MMMI system and developed a corresponding software platform. This system can automatically complete image acquisition, MM computation, and polarization parameter analysis, enabling automated differentiation and providing a detailed analysis of cancerous and normal tissues. This technological platform not only enhances the efficiency and accuracy of data processing but also provides a powerful tool for in-depth studies of the microstructure and polarization characteristics of cancerous tissues.

## 2. Materials and Methods

### 2.1. Schematic Diagram of Full-Automatic MMMI System

**Figure 1:**
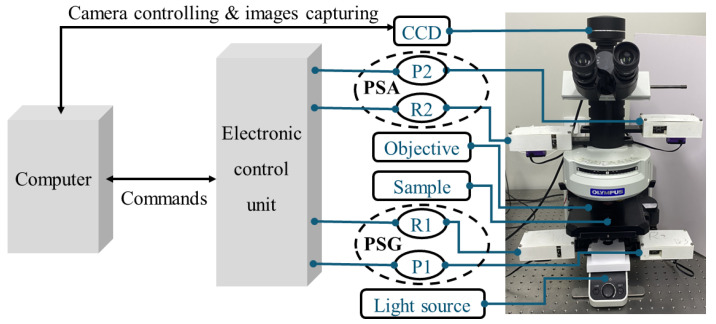
Schematic diagram of MMMI system. P1 and P2, polarizers; R1 and R2, quarter waveplates.

The MMMI system used in this study is modified from a commercial microscope, which is shown in Figure 1. The system primarily comprises a microscope (Olympus, BX53M, Tokyo, Japan) and the polarization modulation modules. The polarization modulation modules include a polarization generator unit and a polarization analyzer unit. The polarization generator unit consists of a linear polarizer (Olympus, U-AN360P, Japan) and a quarter waveplate (manufactured by Union Optics, Wuhan, China), while the polarization analyzer unit consists of a similar quarter waveplate and a linear polarizer. Each optical element within the polarization modulation module is driven to rotate by an electrically controlled servo through a uniform electric control unit, and each polarization modulation module unit is integrated into a designed 3D-printed structure.

In general, the P1 and the R1 are considered as an ensemble that is a polarization state generator (PSG). The light from the light source is modulated firstly by the PSG and then illuminates the sample. On the other hand, the R2 and the P2 construct another ensemble that is called the polarization state analyzer (PSA). The transmission light of the sample is modulated by the PSA and then captured by the camera (Olympus, OHXDP60, 2048 × 3076 pixels).

In what follows, we introduce several main components of our MMMI system, together with the reasons why we selected them.

Modern microscopes use a variety of light sources, and selecting an appropriate light source is crucial depending on the specific microscopy techniques and imaging requirements. Halogen lamps and light emitting diodes (LEDs) are the most common choices for routine imaging [28,29]. Halogen lamps have a continuous spectrum, providing a range from ultraviolet to infrared, and are suitable for both transmitted and reflected light microscopy due to their high brightness. In addition, halogen lamps are often used in conventional brightfield microscopes and differential interference contrast (DIC) microscopes. LEDs are low in power consumption, highly efficient, produce minimal heat, and have a long lifespan and are commonly used in conventional brightfield microscopes and fluorescence microscopes. Specifically, some applications may require LEDs with specific wavelengths. Laser sources, with their excellent monochromaticity and single-wavelength characteristic, are ideal for high-precision imaging, offering very high brightness and the ability to focus on very small spots, but they are costly and require complex optical path designs [30]. Hence, we modified a commercial microscope to build an MM imaging system, using the halogen lamp provided by the microscope as the light source.

Charge-coupled device (CCD) and complementary metal-oxide-semiconductor (CMOS) detectors are the most common choices in modern microscopes [25,31]. CCD detectors offer low noise and high resolution, suitable for applications requiring detailed and clear imaging. CMOS detectors are favored for their fast readout speeds, low power consumption, lower cost, and high integration, making them ideal for fast imaging applications such as live-cell imaging. In our MMMI system, we use a high-resolution CCD camera to obtain clear polarization images of the samples.

Electronic controllers play a crucial role in achieving automation and high-precision control in optical imaging systems. By precisely controlling the rotation angles of polarization modulation modules (including linear polarizers and quarter waveplates), different polarization states of the light source and detection light can be generated for obtaining complete MM data. In the MMMI system, we use servo controllers to achieve high-precision rotation control of the polarization modulation modules through a unified electronic control unit, which ensures the stability and precision of the system, resulting in high-quality imaging data.

This paper employs a low-magnification objective (5×) for MMMI. The low-magnification objective provides a larger field of view, suitable for rapid detection, whereas high-magnification objectives offer higher resolution, suitable for capturing tissue microdetails. Our method ensures detection efficiency while still effectively distinguishing between cancerous and normal tissues.

### 2.2. Measurement of Mueller Matrix

The MM can characterize the linear modulation of light by the sample between the incident light and the transmission light, as shown in Equation (1), where ***M*** represents the MM of the sample, ***M***_P1_ and ***M***_P2_ represents the MM of the linear polarizer, ***M***_R1_ and ***M***_R2_ represents the MM of the quarter waveplate, and ***S*** and ***S’*** represent the Stokes vectors of the incident and the transmission lights, respectively.

(1) $$S^{\prime }=M_{P2}M_{R2}MM_{R1}M_{P1}S,$$

Expanding Equation (1) yields the following:

(2) [S0′S1′S2′S3′]=MP2MR2m11m12m13m14m21m22m23m24m31m32m33m34m41m42m43m44MR1MP1[S0S1S2S3],

where *S*_0_, *S*_1_, *S*_2_, *S*_3_ and *S*’_0_, *S*’_1_, *S*’_2_, *S*’_3_ represent the Stokes parameters of ***S*** and ***S***’, respectively, and *m_ij_* denotes the (*i*, *j*)-th element of ***M***.

To obtain the MM ***M*** of the sample, the MMMI system employs a series of polarized lights generated by altering the azimuth angles of the linear polarizer and the quarter waveplate in the PSG. These polarized lights include the horizontal polarization at 0° (H), the diagonal polarization at 45° (P), the vertical polarization at 90° (V), and the right-handed circular polarization (R), which are used to illuminate the sample. Once the sample is illuminated, the transmission light via the sample is collected by an objective with a magnification factor of 5× and then analyzed by the PSA. In detail, the PSA can be controlled to filter the polarization component of H, P, V, and R out of the transmission light of the sample. These components are finally captured by the camera. Therefore, for each incident polarized light, there are four images captured by the camera, corresponding to intensities of polarization components of H, P, V, and R. In a complete measurement process, the camera captures a total of 16 images for the four types of incident polarized light. For simplicity, we use *I*_HV_ to represent the image captured under the incident polarization state of H and filtering polarization state of V, and based on similar expressions, the images of the MM elements of the sample can be calculated by formulas in Table 1.

### 2.3. Two Kinds of Useful Images Derived from Mueller Matrix Polar Decomposition

The MM contains all the polarization information of the sample, but its physical interpretation for each element is not clear in relation to the sample. On the other hand, MMMI is usually highly sensitive to sample features and related to the microscopic structure. However, considering the fact that the TMA sample is usually quite thin, with the thickness of only 4 μm and thus having the weak scattering, this study adopts the linear phase retardation (*δ*) and the equivalent waveplate fast-axis azimuth angle (*θ*) as two primary parameters to characterize the properties of the sample. Note that both parameters can be derived from the MM polar decomposition [32], and they are well-defined, stable, and with clear physical meanings. Normal tissues contain abundant collagen fibers, which exhibit birefringence and thereby cause significant linear phase retardation (*δ*). Conversely, in cancerous tissues, collagen fibers undergo degradation and destruction, resulting in notably reduced linear phase retardation (*δ*). The equivalent waveplate fast-axis azimuth angle (*θ*) characterizes the angle of the birefringence optical axis; in normal tissues, this axis is relatively orderly distributed, whereas in cancerous tissues, it is comparatively disordered. In fact, similar treatment has been adopted in Ref. [33].

Next, we use the MM polar decomposition to extract polarization parameters with clear physical meanings from the MM. MM polar decomposition provides three main polarization parameters, i.e., the depolarization (Δ), the phase retardance (*R*), and the diattenuation (*D*). The forward polar decomposition of the MM is described by Equation (3), where ***M***_∆_, ***M***_R_, and ***M***_D_ represent the MM corresponding to the depolarizer, the retarder, and the diattenuator, respectively. And ***M***_R_ can be further decomposed into a linear-phase-retarder MM ***M***_LR_ and a circular-phase-retarder MM ***M***_CR_. Note that the linear phase retardance *δ* and the equivalent waveplate fast-axis azimuth angle *θ* of the sample can be calculated by using Equations (4) and (5), respectively, where the lower case ***m***_LR_ is the 3 × 3 sub-matrix of ***M***_LR_, and *ϵ_ijk_* denotes the Levi-Civita permutation symbol, ***m***_LR_(*j*, *k*) represents the elements of ***m***_LR_, and *r*_1_ and *r*_2_ are the vectorial elements of retardance.

(3) $$M=M_{∆}M_{R}M_{D},$$

(4) $$\delta =\cos^{-1}\left\{{\left[\begin{array}{c} {\left(m_{LR}\left(2,2\right)+m_{LR}\left(3,3\right)\right)}^{2}+\\ {\left(m_{LR}\left(3,2\right)+m_{LR}\left(2,3\right)\right)}^{2} \end{array}\right]}^{\frac{1}{2}}\right\}-1,$$

(5) $$\theta =0.5\mathrm{ta}n^{-1}\left(\frac{r_{2}}{r_{1}}\right),r_{i}=\frac{1}{2\sin\delta }\times \sum_{j=1,k=1}^{3}\epsilon_{ijk}m_{LR}\left(j,k\right).$$

In what follows, we will conduct three types of analysis on the images of *δ* and *θ*, extracting relevant parameters to examine the differences and similarities between cancerous and normal samples.

### 2.4. Formatting of Mathematical Components

To analyze the polarization parameter images of samples, the statistics method, the GLCM method, and the TIPM are used in this paper.

Two statistical parameters, i.e., skewness and kurtosis, are widely utilized in biometric detection based on MM [34]. The analysis of the images is performed by calculating kurtosis and skewness, as shown in Equations (6) and (7).

(6) $$Kurtosis=\left(\frac{1}{n}\right){\sum_{i=1}^{n}\left(\frac{x_{i}-x_{mean}}{s}\right)}^{4},$$

(7) $$Skewness=\left(\frac{1}{n}\right){\sum_{i=1}^{n}\left(\frac{x_{i}-x_{mean}}{s}\right)}^{3},$$

where *n* represents the number of samples in the data, *x_i_* denotes the *i*-th data, *x*_mean_ is the mean value of the data, and *s* represents the standard deviation of the data. Xu has pointed out that these two statistical parameters have been widely applied in various fields such as biology [35] and physics [36].

The GLCM is a statistical representation obtained by counting the occurrences of pixel pairs with specific gray-level values at a certain distance in an image. Let *f*(*x*,*y*) be a two-dimensional digital image and *S* be the set of pixel pairs within the target region that exhibit a specific spatial relationship. The GLCM, denoted by ***P***, which captures the occurrences of grayscale values satisfying the defined spatial relationship, can be defined as follows:

(8) $$P\left(i,j\right)=\frac{\#\{[\left(x_{1},y_{1}\right),\left(x_{2},y_{2}\right)]\in S|f\left(x_{1},y_{1}\right)=i\&f(x_{2},y_{2})=j\}}{\#S},$$

where the numerator on the right-hand side of Equation (9) represents the count of pixel pairs with a specific spatial relationship and given grayscale values of *i* and *j*, respectively, while the denominator #*S* represents the total count of pixel pairs. Thus, the profile integration of ***P*** obtained in this way is one.

It should be noted that, although the calculated GLCM cannot be directly applied for texture inspection, texture features can be extracted from it using second-order statistical measures. In this study, parameters including the contrast, the energy, the homogeneity, and the correlation from the GLCM are used for texture analyses of sample images. (1) The parameter of contrast reflects the clarity of an image and the degree of variation in texture. The greater the depth of texture grooves, the higher the contrast, resulting in a clearer visual effect. Conversely, if the contrast is low, the texture grooves appear shallow, resulting in a blurry effect. (2) The parameter of energy is the sum of squared values of the elements in the GLCM, which reflects the degree of uniformity in the grayscale distribution and the coarseness of the texture in an image. Therefore, if all the values in the GLCM are equal, the energy value will be small; if some values are large while others are small, the energy value is large. When the elements in the GLCM are concentrated, the energy value is high. (3) The parameter of homogeneity reflects the roughness of the image texture. Roughness textures have a lower degree of uniformity, while fine textures exhibit a higher degree of uniformity. (4) The parameter of correlation quantifies the similarity between elements of the GLCM in the row or column direction, thereby capturing the local gray-level correlation within an image. The magnitude of the correlation serves as an indicator of the level of local gray-level correlation. A higher correlation is observed when the matrix elements are uniformly distributed and equal. Conversely, a lower correlation is obtained when the matrix elements exhibit significant differences. These parameters can be calculated using Equations (9)–(12).

(9) $$Contrast=\sum_{n=0}^{N_{g}-1}n^{2}\left\{\sum_{i=1}^{N_{g}}\sum_{\begin{array}{c} j=1\\ \left|i-j\right|=n \end{array}}^{N_{g}}P\left(i,j\right)\right\},$$

(10) $$Energy=\sum_{i}\sum_{j}P{\left(i,j\right)}^{2},$$

(11) $$Homogeneity=\sum_{i}\sum_{j}\frac{1}{1+{\left(i-j\right)}^{2}}P\left(i,j\right),$$

(12) $$Correlation=\frac{\sum_{i}\sum_{j}\left(ij\right)P\left(i,j\right)-\mu_{x}\mu_{y}}{\sigma_{x}\sigma_{y}},$$

Here, *N*_g_ represents the number of quantization levels after quantizing the image gray values. *μ_x_*, *μ_y_*, *σ*_x_, and *σ_y_* are the mean and standard deviation of *p*_x_ and *p*_y_, respectively. The GLCM is a method used to describe texture features in digital images. It is based on the spatial relationship between pixel gray values in the image; by analyzing the frequency and positional relationship of different gray levels appearing between pixels, one can extract texture information from the image. Currently, GLCM has been used for the analysis of tumor tissues [37,38].

The TIPM developed by Tamura et al. has gained significant popularity as a powerful approach for selecting optimal image features and designing texture analyzers [39]. To facilitate image description, the TIPM provides a set of quantitative indicators. In this study, we use three textural features, namely, the coarseness, the contrast, and the line-likeness. These parameters can be calculated by using Equations (13)–(15). The parameter of coarseness quantitatively reflects the granularity of texture and is considered as the most fundamental textural feature. When comparing two texture patterns with different elementary scales, the pattern with larger elementary scale is perceived as coarser. The parameter of contrast is derived from the statistical analysis of pixel intensity distribution and is determined by four factors: the grayscale dynamic range, the degree of polarization between the black and white portions on the histogram, the edge sharpness, and the periodicity of repetitive patterns. The parameter of line-likeness is an indicator used to differentiate between line-like and dot-like texture structures.

(13) $$Coarseness=\frac{1}{m\times n}\sum_{i=1}^{m}\sum_{j=1}^{n}S_{best}\left(i,j\right),$$

(14) $$Contrast=\frac{\sigma }{{\left(\alpha_{4}\right)}^{n}},$$

(15) $$Line\text{-}likeness=\frac{\sum_{i=1}^{m}\sum_{j=1}^{n}P\left(i,j\right)\cos\left[\left(i-j\right)\left(\frac{2\pi }{n}\right)\right]}{\sum_{i=1}^{m}\sum_{j=1}^{n}P\left(i,j\right)},$$

Here, *m* and *n* represent the width and height of the image, respectively; *S*_best_ denotes the neighborhood size for maximum intensity similarity; *σ* is the standard deviation of the image; *α*_4_ is the kurtosis of the intensity histogram of the image; *P* is the *m* × *n* local direction co-occurrence matrix; *P*(*i*, *j*) is the element in the *i*-th row and *j*-th column of the matrix *P*. More details about the parameters can be found in the original work [39].

## 3. Results

### 3.1. Original Images of TMA

**Figure 2:**
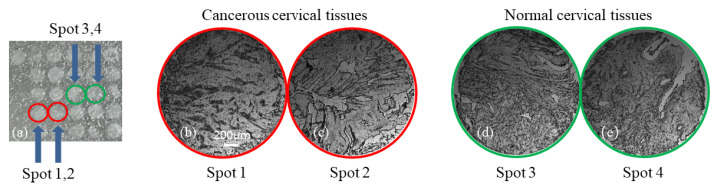
Original images of TMA. (**a**) is the original image of the TMA captured by a general camera; (**b**,**c**) are the original microscopic images of cancerous cervical tissue; (**d**,**e**) are the original microscopic image of normal cervical tissue.

The TMA used in this study is a cancerous cervical and normal cervical tissue composite microarray (provided by Xi’an Zhongke Guanghua, China), where a total of 13 cases are included. Each case consists of two spots: one representing cancerous tissue and the other representing normal tissue. The diameter of each spot is 1.5 mm, and the thickness is 4 μm. The original images of the TMA are shown in Figure 2, where Figure 2a represents the original image of the TMA captured by a general camera, and Figure 2b–e show some samples from the TMA. Figure 2b,c represent two spots of the original microscopic image of cancerous cervical tissue, and Figure 2d,e represent two spots of the original microscopic image of normal cervical tissue. According to the original microscopic images shown in Figure 2b–e, it is challenging to distinguish the cancerous cervical tissue from the normal one.

### 3.2. Mueller Matrix of Cancerous and Normal Cervical Tissues

**Figure 3:**
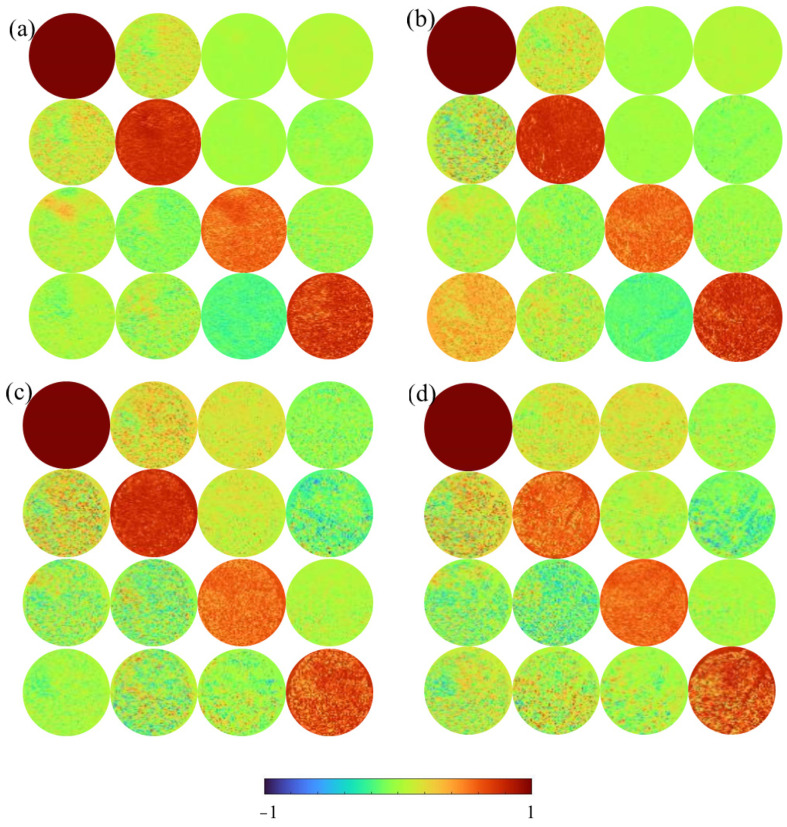
Mueller matrices images of four spots on TMA. (**a**,**b**) are cancerous tissues; (**c**,**d**) are normal tissues.

To accurately distinguish between cancerous and normal tissues, we obtain the MM microscopic images of TMA. Figure 3 shows the MM images of two cancerous cervical tissues (Figure 3a,b) and two normal cervical tissues (Figure 3c,d) in the TMA. We can observe the structural features of the tissues from Figure 3. Compared to cancerous tissues, the *m*_24_, *m*_34_, *m*_42_, and *m*_43_ of normal tissues exhibit more pronounced intensity distributions, which mainly show birefringence. From Figure 3, it can be observed that the values of *m*_22_, *m*_33_, and *m*_44_ are significantly large. This can be attributed to the limited thickness of the tissues, as well as the inverse relationship between the values of *m*_22_, *m*_33_, and *m*_44_ and the depolarization abilities of the tissues. The use of thin tissue samples with a thickness of 4~12 μm for transmission imaging implies limited scattering capability. In this study, the thickness of the samples used is 4 μm, resulting in very small depolarization values for the samples. The values of *m*_12_, *m*_13_, *m*_21_, and *m*_31_, which reflect the diattenuation of the samples, are relatively small. Here, the *m*_12_, *m*_13_, *m*_21_, and *m*_31_ elements of normal tissues show some slightly larger values compared with the cancerous tissues. The MM images demonstrate some differences between the cancerous tissues and the normal tissues shown in Figure 3.

### 3.3. δ and θ Images of Cancerous and Normal Cervical Tissues

**Figure 4:**
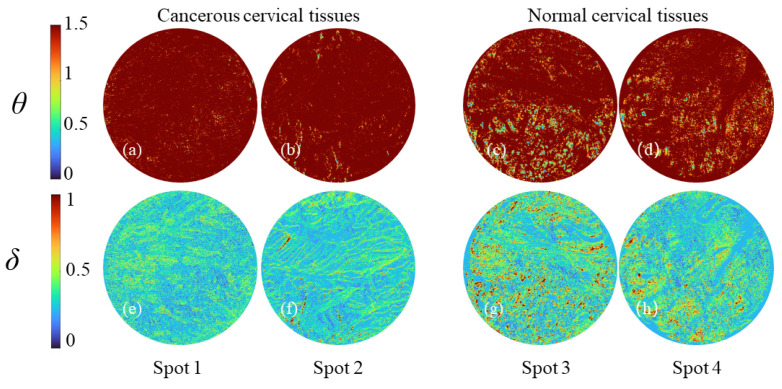
Mueller-matrix-derived polarization parameter images *θ* and *δ* of cancerous cervical tissues and normal cervical tissues. (**a**,**b**,**e**,**f**) are cancerous tissues; (**c**,**d**,**g**,**h**) are normal tissues.

The MM physical interpretation for each element is not clear in relation to the sample, so we adopt the linear phase retarder (*δ*) and the equivalent waveplate fast-axis azimuth angle (*θ*) derived from the MM polar decomposition to characterize properties of the sample. The polarization parameter images of two cancerous cervical tissues and two normal cervical tissues in the TMA are shown in Figure 4, where Figure 4a,b and Figure 4c,d represent *θ* images of the cancerous and the normal cervical tissues, respectively, while Figure 4e,f and Figure 4g,h represent the *δ* images of the cancerous and the normal cervical tissues, respectively. *θ* can characterize the angle of the birefringent optical axis in the sample. The *θ* images of cancerous cervical tissue exhibit a relatively ordered distribution of anisotropic angles throughout the tissue, while the *θ* images of normal cervical tissue show a disordered distribution of anisotropic angles throughout the tissue. Furthermore, from Figure 4g,h, it can be observed that the *δ* value for normal cervical tissue is significantly larger. This is due to the presence of stromal collagen, one of the main components of cervical tissue extracellular matrix, which exhibits birefringence. In cancerous cervical tissue, the stromal collagen is disrupted and degraded, resulting in a significantly smaller *δ* value compared to normal cervical tissue. The two polarization parameters previous research [27] proposed for tissue slice detection can be used in TMA for inspection.

### 3.4. δ and θ Grayscale Images of the Histograms

**Figure 5:**
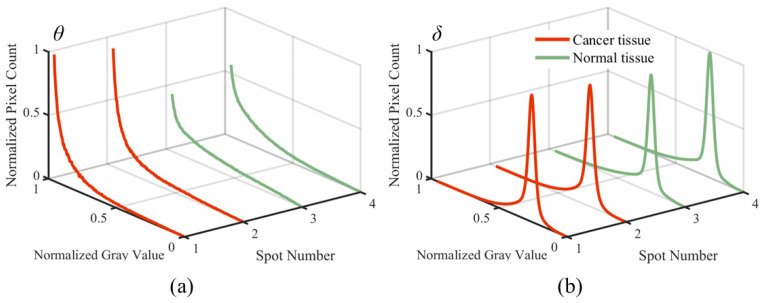
Histograms of grayscale images for *θ* and *δ* parameters. (**a**) is histograms of grayscale images for *θ* parameters; (**b**) is histograms of grayscale images for *δ* parameters.

To further analyze the grayscale image of the spot shown in Figure 2, Figure 5 illustrates the histograms of grayscale images for *θ* and *δ* parameters. The *x*-axis represents spot numbers (1, 2, 3, and 4), the *y*-axis shows normalized grayscale values, and the *z*-axis denotes the normalized pixel count. Spot 1 and 2 correspond to cancerous tissues, while spot 3 and 4 are normal tissues.

Figure 5a displays the histogram of *θ* grayscale images. The grayscale values of cancerous tissues (red curves) predominantly concentrate in the high grayscale region (approximately 0.8 to 1), with normalized pixel counts ranging from 0.5 to 1. This indicates a higher concentration of pixels in the high grayscale range. In contrast, the grayscale values of normal tissues (green curves) are also centered in the high grayscale region (approximately 0.8 to 1), but with a lower peak (normalized pixel count between 0.2 and 0.5). This suggests that cancerous tissues contain a greater number of high grayscale pixels compared to normal tissues.

Figure 5b shows the histogram of *δ* grayscale images. The red curves demonstrate that the grayscale values of cancerous tissues are primarily around 0.7, with normalized pixel counts between 0.4 and 0.45. The green curves indicate that the grayscale values of normal tissues are centered around 0.75, but with a higher peak (normalized pixel count from 0.6 to 1). This implies that cancerous tissues have fewer pixels in the high grayscale region compared to normal tissues.

The analysis of these histograms reveals that although the grayscale value distributions of cancerous and normal tissues are similar for both *θ* and *δ* parameters, there are significant differences in pixel count distributions. Specifically, cancerous tissues exhibit more high grayscale pixels in *θ* grayscale images and fewer high grayscale pixels in *δ* grayscale images. These findings validate the efficacy of our method in distinguishing between cancerous and normal tissues.

### 3.5. Quantitative Analyses of δ and θ Images Using Statistics Method, GLCM, and TIPM

**Figure 6:**
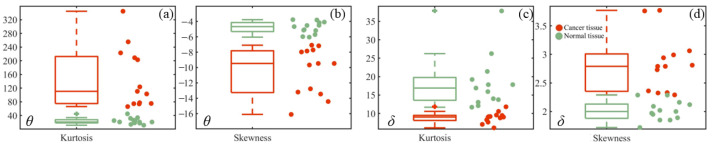
Statistics method parameter boxplots of *θ*, *δ* images. (**a**,**b**) are statistics method parameters boxplots of *θ* images; (**c**,**d**) are statistics method parameters boxplots of *δ* images.

**Figure 7:**
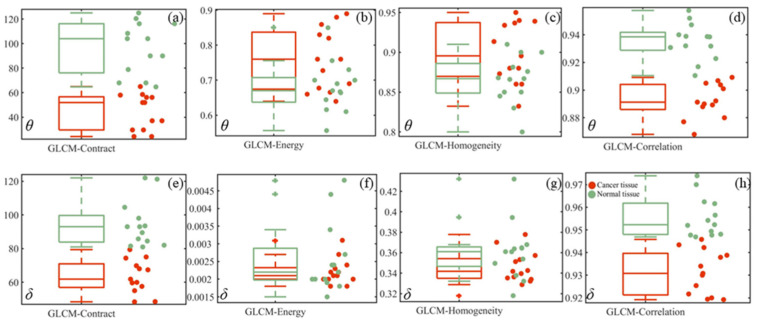
Boxplots of the GLCM parameters for all samples in the TMA. (**a**–**d**) are GLCM parameters boxplots of *θ* images; (**e**–**h**) are GLCM parameters boxplots of *δ* images.

**Figure 8:**
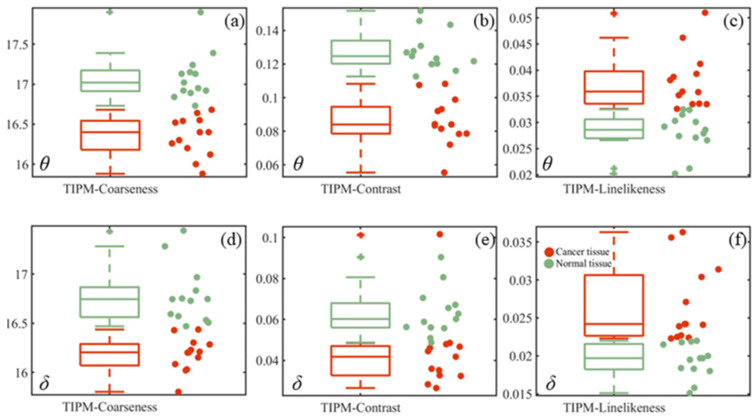
Boxplots of the TIPM parameters for all samples in the TMA. (**a**–**c**) are TIPM parameters boxplots of *θ* images; (**d**–**f**) are TIPM parameters boxplots of *δ* images.

To further analyze the texture features of cancerous and normal tissue images, Figure 6, Figure 7, and Figure 8 present statistics method parameter, GLCM parameter, TIPM parameter boxplots of *θ*, *δ* images, respectively.

Figure 6 presents the boxplots of kurtosis and skewness for 26 samples in the TMA. Figure 6a, b represent the boxplots of *θ*, while Figure 6c,d represent the boxplots of *δ*. In all boxplots, the red dots represent cancerous cervical tissue samples, and the green dots represent normal cervical tissue samples. Figure 6a,d show that the values of cancerous cervical tissues are consistently larger and have a wider distribution compared to that of normal cervical tissues. The kurtosis of *θ* for normal cervical tissues is concentrated around 30, and the median skewness of *δ* is around 2. On the other hand, Figure 6b,c reveal that the values for normal cervical tissues are generally larger than those for cancerous cervical tissues. The skewness values of *θ* for normal cervical tissues are concentrated around −4.5, and the median kurtosis of *δ* is around 17. The kurtosis and skewness of *θ* and *δ* images can effectively differentiate between cancerous cervical tissues and normal cervical tissues.

Figure 7 presents boxplots of the GLCM parameters for all samples on the TMA, with the cancerous cervical tissues indicated by red dots and the normal cervical tissues indicated by green dots. From Figure 7a,e, it can be observed that the contrast parameter of the GLCM reflects the differences in gray levels in the image. The values for cancerous cervical tissues are lower than those for normal cervical tissues, indicating higher contrast in the images of normal cervical tissue. From Figure 7b,f, the overlapping red and green dots suggest similarities in texture features between cancerous cervical tissues and normal cervical tissues. The homogeneity parameter of the GLCM reflects the orderliness of image textures, where smaller values indicate more frequent changes in texture features. In Figure 7c, most of the red dots are distributed above the green dots, indicating that the texture features in the *θ* images of cancerous cervical tissues change more frequently. However, Figure 7g cannot distinguish between cancerous cervical tissues and normal cervical tissues based on this parameter. On the other hand, the correlation parameter of the GLCM reflects the grayscale correlation in the images. As shown in Figure 7d,h, the values for cancerous cervical tissues are lower than those for normal cervical tissues. This is because one of the main components of normal cervical tissue is collagen fibers, which have nearly equal grayscale values in the collagen fiber regions. Therefore, due to the homogeneous internal structure of normal cervical tissue, it exhibits higher autocorrelation. In contrast, the destruction of collagen fibers in cancerous cervical tissues leads to lower autocorrelation compared to normal cervical tissues. From Figure 7a,e,d,h, it is evident that the red and green dots do not overlap. Thus, the contrast and correlation parameters of the GLCM of *θ* and *δ* images can serve as auxiliary diagnostic tools.

Figure 8a–c and Figure 8d–f, respectively, show the boxplots of the Tamura texture features parameters coarseness, contrast and line-likeness for the 26 samples of *θ* and *δ* images. It can be seen from Figure 8a,b,d,e that the coarseness and contrast of normal tissues (green dots) are all higher than those of cancerous tissues (red dots). The line-likeness of cancerous tissues are all larger than normal tissues in Figure 8c,f. From Figure 8, it can be indicated the three parameters can well be used to diagnose cancerous cervical tissues.

## 4. Discussion

The traditional pathological diagnostic process typically requires pathologists to first observe tissue samples using a low-magnification microscope to identify abnormal areas, followed by a high-magnification microscope to determine if the tissue is diseased. This process is not only time-consuming but also extends diagnostic times due to the limited number of pathologists. TMA technology, which consolidates hundreds of different tissue samples onto a single substrate, significantly reduces the need to frequently change slides under the microscope. By understanding these differences, we aim to enhance the efficiency of cancer diagnostics.

We have developed an automated MMMI system capable of capturing TMA samples and performing MM decomposition. This system captures images of cancerous cervical TMA using a low-magnification objective (5×) and directly uses MM parameters for diagnosis, eliminating the need to switch to a high-magnification objective and thus saving substantial time.

Unlike traditional MM microscopic techniques that use high-magnification objectives (20× to 50×) [40,41], our study focuses on analyzing the MM parameters of pathological tissues under a low-magnification objective (5×) specific to TMA characteristics. It should be noted that high-magnification objectives (20×–50×) provide high-resolution imaging with a small field of view, whereas a low-magnification objective (5×) offers low-resolution imaging with a large field of view. This implies that the polarized images under high magnification due to their high resolution can reveal more detailed information, thereby allowing more accurate differentiation between cancerous and normal tissues. However, the process is less efficient as it requires initially using a low-magnification objective to determine the range of abnormal tissues, followed by gradual switching to high-magnification objectives to delineate the diseased and non-diseased areas. Although a low-magnification objective provides less detailed information compared to high-magnification objectives, according to the data in Table 2, the information contained is sufficient to distinguish between cancerous and normal tissues. Furthermore, due to the larger field of view of a low-magnification objective, a more rapid diagnosis of cancerous tissues can be achieved. The data presented in Table 2 clearly show the differences between cancerous and normal tissues in the TMA, providing critical auxiliary diagnostic data for postoperative biopsies.

To further illustrate the differences between low- and high-magnification objectives in MMMI, Table 3 presents the variation in the *θ* images and *δ* images mean gray values measured using 5×, 10×, 20×, and 50× objectives. It is found that the differences in mean gray values of *θ* and *δ* between the cancerous image and the normal image increase as the magnification increases. This is because higher-magnification objectives offer higher resolution, capturing more tissue details. However, even for a 5× objective, the differences are still sufficient for distinguishing normal and cancerous tissues.

The high-throughput TMA and low-magnification objectives (5×) used in this study have clear advantages in rapid diagnosis, particularly in large-scale screening and initial examination. Nonetheless, rapid diagnosis may impact the accuracy and sensitivity of the diagnosis to some extent. Low-magnification objectives, while providing a larger field of view beneficial for rapid scanning and detection, have lower resolution, which may lead to the insufficient identification of certain subtle pathological features, thereby affecting diagnostic accuracy and sensitivity. Cancer cells can be classified into stages I, II, III, and IV based on their differentiation; they can be precisely distinguished under high-magnification objectives, but under low-magnification objectives, only the distinction between cancerous tissues and normal tissue is achieved but not differentiation between different stage of cancerous tissues.

For further discussion, we have provided a summary comparison Table 4 on the recently reported literature with Limit of detection (LOD), sensitivity, measurement error, and application. Classifying MM microscopy techniques, they can be divided into two major categories: (1) MM microscopy explores the polarization characteristics of unstained pathological slices, aiming to diagnose different stages of unstained pathological sections through polarization parameters, and therefore does not report sensitivity. Number 1 [40] of the Table 4 demonstrates that the depolarization, the equivalent waveplate fast-axis azimuth angle, and the phase retardation parameters can differentiate normal colon tissue, stage II, and stage III colon cancer. Number 2 [42] of the Table 4 shows that depolarization, the equivalent waveplate fast-axis azimuth angle and the phase retardation, and anisotropy parameters can distinguish between stages I to IV of Breast ductal carcinoma. (2) MM microscopy explores the polarization characteristics of stained slices. Number 3 [41] of the table utilizes 16 MM elements combined with deep learning to diagnose giant cell tumor, achieving a sensitivity of 99.45%. Number 4 [43] of the table combines MM elements with deep learning techniques to diagnose mouse skin cancer, achieving a sensitivity of 94%. Table 4 summarizes the above data and lists the magnification and measurement error used in each work.

TMA technology has been widely applied, for instance, in placental research, to achieve high-throughput tissue analysis [11]. However, existing studies often employ tissue staining or immunohistochemistry, which require specific probes and stringent experimental conditions. In contrast, our combination of TMA technology with MMMI achieves label-free, efficient detection for the first time, significantly enhancing the clinical applicability of TMA.

Despite demonstrating a highly reliable method for detecting cervical cancer, our study has some limitations. First, the sample including 13 cases of cancerous cervical and normal cervical tissues is relatively small, potentially affecting statistical significance. Future studies should increase the sample size to verify the generalizability of our results. Second, the TMA samples in this study are 4 μm thick, which may introduce biases. Future research should explore the impact of different sample thicknesses on detection results.

This study aims to provide an efficient postoperative pathological diagnostic method. The combination of MMMI and TMA technology shows great potential in tissue detection and cancer diagnosis. Future studies could further apply this technology to other cancer types (such as breast cancer and prostate cancer) and high-throughput tissue analysis platforms (such as pathological slide scanning) to improve clinical diagnostic efficiency. Optimizing system configuration and image analysis algorithms will also help enhance detection sensitivity and specificity.

In summary, this study combines MMMI and TMA technology to achieve efficient detection of cancerous cervical tissue. The results indicate that linear phase retardance (*δ*) and effective waveplate fast-axis orientation (*θ*) are effective parameters for distinguishing cancerous from normal tissues. While there is still room for improvement, our preliminary results provide a new technical pathway for postoperative pathological diagnosis and offer key references for future research directions.

## 5. Conclusions

TMA technology has the potential to significantly enhance the efficiency of sample preparation, thereby reducing the postoperative biopsy histopathological diagnosis time. The MMMI technique can overcome the limitations of specific probes, storage conditions, and fluorescent labeling requirements, enabling the label-free, non-destructive, and non-contact detection of TMA inspection. In this study, we employed TMA in conjunction with the MMMI technique for cancerous cervical detection. The two polarization parameters by using a high-magnification objective (20×–50×), namely the linear phase retardance equivalent waveplate fast-axis azimuth *θ* and linear phase retardance *δ* images, which were previously proposed for tissue slice detection, can also be used for TMA inspection. Statistics methods, GLCM, and TIPM were utilized to extract features from these polarization parameter images. The experimental and analytical results obtained using the low-magnification objective (5×) indicate that the *θ* and *δ* images can serve as effective parameters for distinguishing the polarization microscopy image features of cancerous cervical tissues from normal cervical tissues. These findings highlight the significant value of the proposed approach for the histopathological diagnosis of cancer in postoperative biopsies, as well as the detection of tissue microarrays (TMAs).

## Figures and Tables

**Table 1:** Formulas of the Mueller matrix elements of the sample.

Element	Formula
*m*_11_	$I_{HH}+I_{HV}+I_{VH}+I_{VV}$
*m*_12_	$I_{HH}+I_{HV}-I_{VH}-I_{VV}$
*m*_13_	$2I_{PH}+2I_{PV}-I_{HH}-I_{HV}-I_{VH}-I_{VV}$
*m*_14_	${2I}_{RH}+{2I}_{RV}-I_{HH}-I_{HV}-I_{VH}-I_{VV}$
*m*_21_	$I_{HH}-I_{HV}+I_{VH}-I_{VV}$
*m*_22_	$I_{HH}-I_{HV}-I_{VH}+I_{VV}$
*m*_23_	${2I}_{PH}-2I_{PV}-I_{HH}+I_{HV}-I_{VH}+I_{VV}$
*m*_24_	${2I}_{RH}-{2I}_{RV}-I_{HH}+I_{HV}-I_{VH}+I_{VV}$
*m*_31_	${2I}_{HP}+{2I}_{VP}-I_{HH}-I_{HV}-I_{VH}-I_{VV}$
*m*_32_	${2I}_{HP}-{2I}_{VP}-I_{HH}-I_{HV}+I_{VH}+I_{VV}$
*m*_33_	${4I}_{PP}-2I_{PH}-2I_{PV}-{2I}_{HP}-{2I}_{VP}+I_{HH}-I_{HV}-I_{VH}-I_{VV}$
*m*_34_	${4I}_{RP}-2I_{RH}-2I_{RV}-{2I}_{HP}-{2I}_{VP}+I_{HH}-I_{HV}-I_{VH}-I_{VV}$
*m*_41_	${2I}_{HR}+{2I}_{VR}-I_{HH}-I_{HV}-I_{VH}-I_{VV}$
*m*_42_	${2I}_{HR}-{2I}_{VR}-I_{HH}-I_{HV}+I_{VH}+I_{VV}$
*m*_43_	$4I_{PR}-{2I}_{PH}-2I_{PV}-{2I}_{HR}-{2I}_{VR}+I_{HH}+I_{HV}+I_{VH}+I_{VV}$
*m*_44_	${4I}_{RR}-2I_{RH}-2I_{RV}-{2I}_{HR}+{2I}_{VR}+I_{HH}+I_{HV}-I_{VH}-I_{VV}$

**Table 2:** Values of grayscale, statistics method parameters, GLCM parameters, and TIPM parameters for *θ* and *δ* images.

		*θ* Images		*δ* Images	
		Cancer	Normal	Cancer	Normal
	High grayscale normalized pixels	0.5–1	0.2–0.5	0.4–0.45	0.6–1
Median value of statistics method parameters	Kurtosis	200	30	−8	17
	Skewness	−12	−4.5	3	2
Median value of GLCM parameters	Contrast	40	90	40	100
	Energy	0.76	0.67	0.0022	0.0023
	Homogeneity	0.89	0.87	0.34	0.345
	Correlation	0.89	0.930	0.93	0.952
Median value of TIPM parameters	Coarseness	16.4	17	16.2	16.7
	Contrast	0.082	0.122	0.041	0.006
	Line-likeness	0.036	0.028	0.024	0.019

**Table 3:** Mean gray values of *θ* and *δ* images.

Objective	*θ* Images			*δ* Images		
	Cancer	Normal	Difference	Cancer	Normal	Difference
5×	251	235	16	78	90	12
10×	251	220	31	70	100	30
20×	253	200	53	69	110	41
50×	254	190	64	60	130	70

**Table 4:** LOD, sensitivity, measurement error, and application of Mueller matrix microscopic imaging under different magnifications.

Number	Method	LOD	Sensitivity	Measurement Error	Application
1	25× objective	stage II	/	6%	Colon cancer
2	10× objective	stage I	/	About 1%	Breast ductal carcinoma
3	40× objective and deep learning	Distinguish between normal and abnormal tissues	99.45%	About 1%	Giant cell tumor of bone
4	100× objective and deep learning	Distinguish between normal and abnormal tissues	94%	About 1%	Mice non-melanoma skin cancer

## Data Availability

The data and materials information that support the findings of this study are available from the corresponding author upon reasonable request.

## Abbreviations

Abbreviations	Full name
MMMI	Mueller matrix microscopic imaging
TMA	Tissue microarray
MM	Mueller matrix
GLCM	Gray-level co-occurrence matrix analysis
TIPM	Tamura image processing method
HPV	Human papilloma virus
PCR	Polymerase chain reaction
DIC	Differential interference contrast
LED	Light emitting diode
CCD	Charge-coupled device
CMOS	Complementary metal oxide semiconductor
PMT	Photomultiplier tube
APD	Avalanche photodiode
*θ*	The equivalent waveplate fast-axis azimuth angle
*δ*	The phase retardation
PSG	Polarization state generator
PSA	Polarization state analyzer
H	The horizontal polarization at 0°
P	The diagonal polarization at 45°
V	The vertical polarization at 90°
R	The right-handed circular polarization
*D*	Diattenuation
Δ	Depolarization
*R*	Phase retardance
L	Linear
C	Circular
LOD	Limit of detection
